# Growth Arrest-Specific 6 in Chromophobe Renal Cell Carcinoma

**DOI:** 10.1159/000525601

**Published:** 2022-06-27

**Authors:** Marie Mikuteit, Stefanie Zschäbitz, Maximilian Erlmeier, Michael Autenrieth, Wilko Weichert, Arndt Hartmann, Sandra Steffens, Franziska Erlmeier

**Affiliations:** ^a^Department for Rheumatology and Immunology, Hannover Medical School, Hannover, Germany; ^b^Dean's Office − Curriculum Development, Hannover Medical School, Hannover, Germany; ^c^Department of Medical Oncology, National Center of Tumor Diseases, University Hospital Heidelberg, Heidelberg, Germany; ^d^Department of Urology, München Klinik Bogenhausen, Munich, Germany; ^e^Department of Urology, Technical University of Munich, Klinikum rechts der Isar, Munich, Germany; ^f^Institute for Pathology and Pathological Anatomy, Technical University Munich, Munich, Germany; ^g^Member of the German Cancer Consortium (DKTK), Heidelberg, Germany; ^h^Institute of Pathology, University Hospital of Erlangen, Erlangen, Germany; ^i^Department of Urology, University Hospital Münster, Münster, Germany

**Keywords:** Renal cell carcinoma, Growth arrest-specific protein 6, Chromophobe histology, Survival

## Abstract

**Background:**

Overexpression of tumor-associated growth arrest-specific protein 6 (Gas6) is found in many tumor entities. The prognostic value of Gas6 in renal cell carcinoma (RCC), especially in non-clear cell RCC, is still unclear.

**Aim:**

The aim of the study was to evaluate the prognostic impact of Gas6 expression in a large cohort of patients with chromophobe RCC (chRCC).

**Material and Methods:**

Patients who underwent renal surgery due to chRCC were retrospectively evaluated. Tumor specimens were analyzed for Gas6 expression by immunohistochemistry.

**Results:**

Eighty-one chRCC patients were eligible for analysis; of these, 24 (29.6%) patients were positive for Gas6. No significant associations were found for Gas6 expression and clinical attributes in patients with chRCC. The Kaplan-Meier analysis revealed no differences in 5-year overall survival for Gas6− compared to Gas6+ (89.6% vs. 100.0%; *p* = 0.288) tumors.

**Conclusion:**

In chRCC, Gas6 expression is not associated with survival and other parameters of aggressiveness. Due to the rare incidence of chRCC, further studies with larger cohorts are warranted.

## Introduction

Growth arrest-specific protein 6 (Gas6) is a vitamin k-dependent ligand of TAM (Tyro3, Axl, and MER receptor tyrosine kinases [1]). The Gas6/TAM ligand-receptor complex transmits signals from the extracellular to the intercellular space and plays an important role in cell proliferation. It was shown that Gas6 is expressed in many tumor types, such as pancreatic and ovarian cancer, melanoma, and leukemia [2, 3, 4, 5]. The presence of Gas6/TAM inhibits cell death and leads to proliferation of tumor cells [6]. Also, Gas6/TAM promotes migration of the tumor cells and, thus, metastasis [7, 8]. In some cancer types, it could be demonstrated that a higher expression of Gas6 and Axl were associated with poorer survival [4].

Also, in renal cell carcinoma (RCC), Gas6 expression is found. Most studies measured Gas6 in clear cell RCC (ccRCC), but there are smaller cohorts of non-ccRCC [9]. Additionally, expression of Gas6 was associated with poorer survival [10]. Rankin et al. [11] showed that in metastatic ccRCC, the activation of the AXL/Gas6 pathway was associated to a higher mortality. Since RCC is not sensitive for chemotherapy, targeted therapies are warranted. Hakozaki et al. discuss Gas6/Axl to be a target in RCC treatment [9].

Chromophobe RCC (chRCC) is less common than ccRCC. With 5–7% of all RCC, it is the third most common type. The RCC type is less aggressive than other ones and the outcome is generally good with a 5-year survival rate of 78–100% [12]. Nevertheless, some patients develop metastases, most commonly in liver and lungs. In rare cases, chRCC is associated with the genetic disease Birt-Hogg-Dubé syndrome. Up to now, there is no valid grading system for this tumor subtype. Furthermore, no prognostic biomarkers exist. To the best of our knowledge, this study is the first to systematically evaluate the prognostic value of Gas6 in chRCC in a large cohort.

## Material and Methods

### Patients and Tumor Characteristics

Eighty-one patients, who underwent renal surgery for chRCC between 1996 and 2014, were identified using the electronic pathology register. All patients have been treated at the Department of Urology, Technical University of Munich, Klinikum rechts der Isar. Clinical attributes relating to each tissue sample were collected among those tumor stage and histological subtype according to the UICC 2010 TNM tumor staging system. Suitable specimens were selected by a pathologist (F.E.), and tissue micro arrays (TMA) were prepared from the primary tumor as previously described [13]. The histological subtype was confirmed by a second uropathologist (A.H.). Patient data were retrieved from electronic patient charts, with follow-up data regarding overall survival (OS) and death being ascertained from the Munich Cancer Registry of the Munich Tumor Center. The study was approved by the Ethics Committee of the Technical University of Munich (384/13) in accordance with the German Human Research Act and with the Declaration of Helsinki.

### Procedures

Expression of Gas6 was determined by immunohistochemistry (IHC). Therefore, 2 mm TMA slides were stained for Gas6 (Anti-Gas6 antibody, R&D Systems, AF885, dilution 1:20) with a fully automated Dako Autostainer (Dako, Agilent pathology systems). Antigen retrieval was accomplished at pH 7.2. For visualization of bound primary antibody, EnVision Detection Kit (Dako, EnVision+ System-HRP) was used. Sections were rinsed in tap water, counterstained with Mayer's Hematoxylin solution, and finally mounted. Images were captured under a Leitz ARISTOPLAN light microscope (Leica Microsystems, Germany) with a ×10 eyepiece, a 22-mm field of view and a ×40 objective lens (Plan FLUOTAR x40/0.70). Tissue sections were analyzed in a blind way by a pathologist (F.E.). Paraffin-embedded human seminal vesicle tissue was used as the positive control.

The staining reaction was classified according to a semiquantitative IHC reference scale previously described [14, 15]. Gas6 was localized primarily on the membrane and partly in the cytoplasm of tumor cells.

The staining intensity was scored from 0 to 3 (0 = no staining, 1 = weak staining, 2 = moderate staining, 3 = strong staining) according to the H-Score as already described [16, 17, 18]. The area of staining was evaluated in percent (0–100%); a staining intensity score was defined by multiplying the score with the stained area [19, 20, 21]. Given the absence of normative data on cell membrane or cell cytoplasm staining intensity in the literature, values in our patient collective were dichotomized using the median of observed distribution as the cutoff. Because of the limited number of cases, a binary cutoff was used. A Gas6 staining lower to the median was defined as Gas6^-^, and a staining higher or equal to the median was defined as Gas6^+^.

### Statistical Analysis

The primary endpoint of the study was OS. In the absence of death, the endpoint was censored at the last date of follow-up. The duration of follow-up was calculated from the date of surgery to the date of death or last known follow-up. Dependent upon the nature of variable, χ^2^, Fisher's exact tests, Mann-Whitney U test, and independent *t* test were used as appropriate, to compare between patient/tumor characteristics and the corresponding subgroup with or without Gas6 expression. Kaplan-Meier survival times were estimated, with subgroups being compared using the log-rank test. SPSS 27.0 (USA) was used for statistical assessment. Two-sided *p* values below 0.05 were considered statistically significant.

## Results

### Patients' Characteristics and Gas6 Expression

**Fig. 1:**
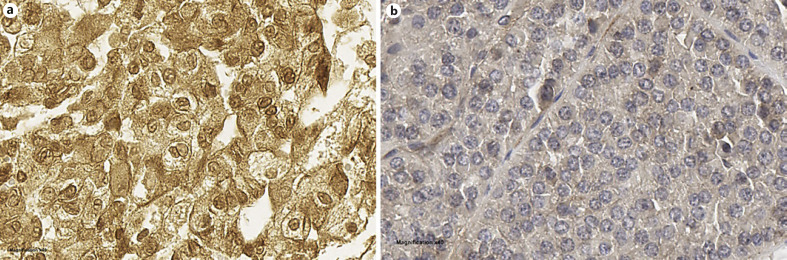
Immunohistochemical staining of Gas6 in chromophobe RCC specimen. **a** Positive (×40 magnification). **b** Negative (×40 magnification).

The median age of the cohort was 59.8 (range: 31–79) years. Sixty patients (74.1%), 14 patients (17.3%), and 7 patients (8.6%) presented with pT1, pT2, and pT3 tumors, respectively. 86.4% of the patients had AJCC Stage I/II. Furthermore, 6 (7.4%) of all patients presented with lymph node metastasis and/or synchronous distant metastasis. Gas6 expression was found in 49 (60.5%) of the chRCC TMA specimens (Fig. 1). No associations between Gas6 expression and patient or tumor characteristics were identified (Table 1).

### Gas6 Expression and Clinical Course

**Fig. 2:**
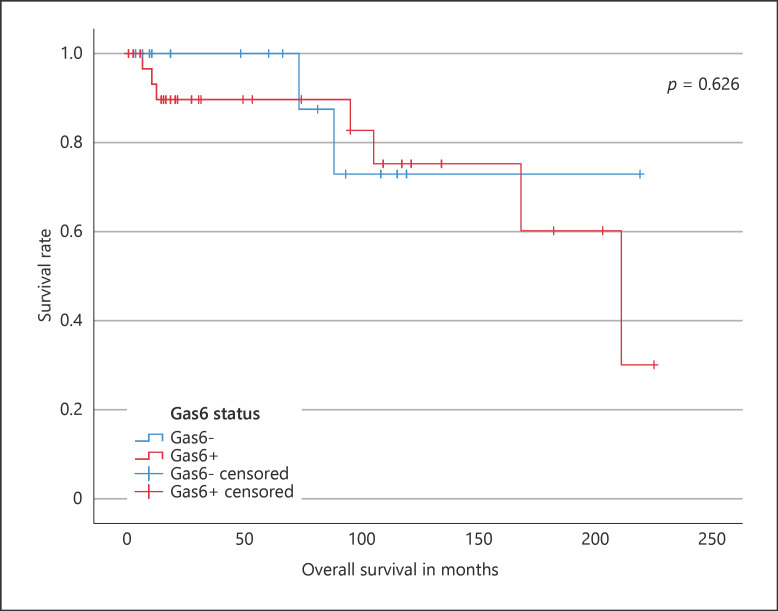
The Kaplan-Meier analysis disclosed an OS for Gas6− compared to Gas6+ tumors of 100.0% compared to 89.7% (*p* = 0.626, log rank).

Median follow-up was 40.5 (IQR: 10.8–109.3) months. At the time of last follow-up, 46 (56.8%) patients were alive, 9 (43.5%) patients died and 26 (32.1%) patients were lost to follow up. In the group of patients with Gas6− and Gas+ tumors, 18 (56.3%) and 28 (57.1%) patients were alive, 2 (6.3%) and 7 (14.3%) patients died and 12 (37.5 and 14 (28.6%) were lost to follow-up, respectively (*p* = 0.447, χ^2^). The Kaplan-Meier analysis disclosed an OS for Gas6− compared to Gas6+ tumors of 100.0% compared to 89.7% (*p* = 0.626, log rank; Fig. 2).

## Discussion

Gas6 and TAM play an important role in prognosis in different tumor types since higher expression of Gas6 is associated with poorer survival [4]. Patients with pancreatic cancer and a higher Axl expression had more often affected lymph nodes and showed a shorter median survival (12 vs. 18 months, *p* < 0.001 [22]). The expression of Axl is also a negative prognostic factor in the survival of early-stage colon cancer patients [23]. Contrary, in breast cancer patients Gas6 was not associated with poorer survival [24]. Gustafsson et al. [10] showed that an association between Gas6 expression and poorer survival appears also in their RCC cohort (all types). Five-year survival for 12 chRCC patients was 86%, which is comparable to our results [10]. Gas6/Axl also induces cellular invasion through the MET/SRC pathway [11]. It was also shown that Gas6 scores were higher in chRCC tumors compared to papillary RCC, and even higher in metastases of all RCC types [9]. The aim of our study was to evaluate Gas6 as prognostic factor in a cohort of 81 patients with chRCC. We could not find a significant association of any tumor or patient specific characteristics and expression of Gas6. Also, we could not show a significant difference in OS in Gas6+ versus Gas6− tumor patients. The relevance of Gas6 in prognosis of chRCC tumor patients needs further investigation.

Apart from the prognostic function of Gas6, the molecule can be discussed as possible target in RCC treatment. Higher Gas6/Axl expression was associated with less effectiveness of tyrosine kinase inhibitor (TKI) therapy [9]. Some TKIs also suppress the Gas6/TAM axis and are available with limited indications, but there is no specific TKI for Gas6/TAM [25]. Also, multikinase inhibitors, such as cabozantinib are highly effective in RCC treatment [26]. However, in chRCC efficacy seems to be lower compared to other RCC subtypes [27, 28]. In breast cancer patients, expression of Gas6 was associated with a resistance against the treatment [29, 30] or less effectiveness of TKI therapy [9]. Gas6 is also discussed as a possible marker for the efficacy of a treatment. Gas6/Axl expression was higher in patients with residual ovarian cancer [4] or in metastatic ccRCC patients [11]. But also, the downregulation of Gas6 and TAM can cause problems since it is associated with autoimmunity [31]. The role of Gas6 can vary in different cancer types and depends on the gene regulation [32]. Further research should investigate the role of gene regulation of Gas6 and TAM in tumor progress and autoimmunity.

To our knowledge, this is the first study that assesses the prognostic role of Gas6 in a larger cohort of 81 patients with chRCC. But our study also has limitations. The number of analyzed patients is still relatively small due to the small incidence of this tumor type. Also, the methods of immunohistochemical and retrospective analysis lead to results that should be interpreted with caution.

In conclusion, in chRCC, Gas6 expression is not associated with survival and other parameters of aggressiveness in our cohort. However, as Gas6 showed a high potential as prognostic and therapeutic marker in several tumor entities. Further studies, which focus on non-clear cell RCC, are needed.

## Statement of Ethics

The study was approved by the Ethics Committee of the Technical University of Munich (384/13) in accordance with the German Human Research Act and with the Declaration of Helsinki. Informed written consent was obtained from all individual participants included in the study. Details that disclose the identity of the subjects under study were omitted.

## Conflict of Interest Statement

All the authors declare that they have no competing interests.

## Funding Sources

This work was supported by a grant of the Deutsche For­schungsgemeinschaft (DFG), GZ:ER 795/1-1 (Franziska Erlmeier).

## Author Contributions

Marie Mikuteit, Franziska Erlmeier, and Sandra Steffens participated in the data interpretation and drafting of the manuscript. Marie Mikuteit performed the statistical analysis. Franziska Erlmeier carried out the data acquisition. Stefanie Zschäbitz, Max Erlmeier, Michael Autenrieth, Wilko Weichert, and Arndt Hartmann revised the manuscript for important intellectual content. All the authors read and approved the final manuscript.

## Data Availability Statement

The datasets generated during and/or analyzed during the current study are available from the corresponding author on reasonable request.

## Figures and Tables

**Table 1:** chRCC patients' and tumor characteristics in dependence of Gas6 expression

Variable	All chRCC, *n* = 81 (100%)	Gas6^−^, *n* = 32 (39.5%)	Gas6^+^, *n* = 49 (60.5%)	*p* value
Age, median (IQR), years	59.8 (52.0–69.1)	59.6 (50.5–67.9)	61.9 (52.4–69.9)	0.595^a^
Sex, *n* (%)				
Female	23 (28.4)	7 (21.9)	16 (32.7)	0.325^b^
Male	58 (71.6)	25 (78.1)	33 (67.3)	
Stage (TNM 2010), *n* (%)				
pT1	60 (74.1)	25 (78.1)	35 (71.4)	0.759^c^
pT2	14 (17.3)	5 (15.6)	9 (18.4)	
pT3	7 (8.6)	2 (6.3)	5 (10.2)	
Cancer stage (AJCC), *n* (%)				
Stage I	56 (69.1)	24 (75.0)	17 (70.8)	0.777^c^
Stage II	14 (17.3)	5 (15.6)	9 (18.4)	
Stage III	8 (9.9)	2 (6.3)	6 (12.2)	
Stage IV	3 (3.7)	1 (3.1)	2 (4.1)	
LN metastasis*,^#^ n* (%)				
N–	78 (96.3)	32 (100)	46 (93.9)	0.274^b^
N+	3 (3.7)	0 (0.0)	3 (6.1)	
Metastasis*,^#^n* (%)				
M–	78 (96.3)	31 (96.9)	47 (95.9)	1.0^b^
M+	3 (3.7)	1 (3.1)	2 (4.1)	
Disease status, *n* (%)				
Localized*	70 (86.4)	29 (90.6)	41 (83.7)	0.513^b^
Advanced^$^	11 (13.6)	3 (9.4)	8 (16.3)	

IQR, interquartile range; LN, lymph node; N–, lymph node status unknown or tumour cells absent from regional lymphnodes; N+, regional lymph node metastasis present.

# At time of renal surgery.

* Localized disease = pT1/2 N0/M0.

$ Advanced disease = pT3/4 and/or N+ and/or M+.

a Mann-Whitney U test.

b Fisher's exact test.

c χ^2^ test.
